# Genogeography and Immune Epitope Characteristics of Hepatitis B Virus Genotype C Reveals Two Distinct Types: Asian and Papua-Pacific

**DOI:** 10.1371/journal.pone.0132533

**Published:** 2015-07-10

**Authors:** Meta Dewi Thedja, David Handojo Muljono, Susan Irawati Ie, Erick Sidarta, Turyadi, Jan Verhoef, Sangkot Marzuki

**Affiliations:** 1 Eijkman Institute for Molecular Biology, Jakarta, Indonesia; 2 Eijkman Winkler Institute, University Medical Centre (UMC) Utrecht, Utrecht, The Netherlands; 3 Faculty of Medicine, Hasanuddin University, Makassar, Indonesia; 4 Sydney Medical School, University of Sydney, Sydney, New South Wales, Australia; 5 Department of Medicine, Monash Medical Centre, Monash University, Clayton, Victoria, Australia; Singapore Institute for Clinical Sciences, SINGAPORE

## Abstract

Distribution of hepatitis B virus (HBV) genotypes/subgenotypes is geographically and ethnologically specific. In the Indonesian archipelago, HBV genotype C (HBV/C) is prevalent with high genome variability, reflected by the presence of 13 of currently existing 16 subgenotypes. We investigated the association between HBV/C molecular characteristics with host ethnicity and geographical distribution by examining various subgenotypes of HBV/C isolates from the Asia and Pacific region, with further analysis on the immune epitope characteristics of the core and surface proteins. Phylogenetic tree was constructed based on complete HBV/C genome sequences from Asia and Pacific region, and genetic distance between isolates was also examined. HBV/C surface and core immune epitopes were analyzed and grouped by comparing the amino acid residue characteristics and geographical origins. Based on phylogenetic tree and geographical origins of isolates, two major groups of HBV/C isolates—East-Southeast Asia and Papua-Pacific—were identified. Analysis of core and surface immune epitopes supported these findings with several amino acid substitutions distinguishing the East-Southeast Asia isolates from the Papua-Pacific isolates. A west-to-east gradient of HBsAg subtype distribution was observed with *adrq*+ prominent in the East and Southeast Asia and *adrq*- in the Pacific, with several *adrq*-indeterminate subtypes observed in Papua and Papua New Guinea (PNG). This study indicates that HBV/C isolates can be classified into two types, the Asian and the Papua-Pacific, based on the virus genome diversity, immune epitope characteristics, and geographical distribution, with Papua and PNG as the molecular evolutionary admixture region in the switching from *adrq*+ to *adrq*-.

## Introduction

Worldwide, an estimated two billion people have been infected with hepatitis B virus (HBV) and more than 240 million have chronic liver infections. About 780,000 people die every year due to the acute or chronic consequences of hepatitis B [1]. In endemic regions such as Asia and Pacific where most infections occur perinatally or in early childhood, up to 15–40% of individuals with chronic hepatitis B (CHB) will progress to cirrhosis, end-stage liver disease, or hepatocellular carcinoma (HCC) during their lifetime [2]. HBV genetic variations, e.g. genotype and subtype, and mutations in some regions have been associated with diagnostic problems, failure to hepatitis B vaccination, and different clinical manifestations such as development of cirrhosis and HCC, and response to treatment [3–6].

HBV has been classified into 9 genotypes (A to I) and one ‘putative’ genotype (J) based on the divergence over the entire genome [4,7–12]. Based on some antigenic determinants of the surface antigen (HBsAg), nine serological types, referred to as subtypes—*adw2*, *adw4*, *adrq+*, *adrq-*, *ayw1*, *ayw2*, *ayw3*, *ayw4* and *ayr*—have been identified [13,14]. HBV genotypes and subtypes have a distinct geographical distribution worldwide, parallel to and presumably evolve in populations of different ethnic origins [9,15].

In Asia and Pacific islands, HBV genotype B (HBV/B) and genotype C (HBV/C) are the predominant genotypes. Compared to HBV/B, HBV/C is more often associated with higher rates of hepatitis B *e* antigen (HBeAg) carriers, lower rates of spontaneous HBeAg seroconversion, higher HBV DNA levels, with higher histological activities and higher proportion of patients developing cirrhosis and HCC [16–18]. In Indonesia, HBV/C is largely found in populations of the eastern islands, mostly in agreement with *adrq+* and *adrq*-indeterminate subtype distribution [19], while HBV/B is typical in populations of the western islands of Indonesia, in parallel with the distribution of subtype *adw* [20,21].

HBV/C has been classified into sixteen subgenotypes, C1 to C16, each with specific geographical distribution. C1 (Cs) and C2 (Ce) were found predominantly in two different regions: C1 in Southeast Asia and C2 in east Asia [15,22,23]. C3 was found in the Oceania [15], C4 in Australian Aborigines [24], with C5 and C7 in the Philippines [25,26]. Six other subgenotypes, C6, C8, C9, C10, C11, C12, and the recently reported C13, C14, C15, and C16 were found in the Indonesian archipelago [19,27–29]. These ten subgenotypes were distinctly distributed: C6 in isolated populations of part of Papua, C8 in Nusa Tenggara and some western part of Indonesia (Denpasar, Jakarta, Banjarmasin, and Palembang), C9 in Timor Leste, and C10 in Nusa Tenggara, while C11-16 were found in Papua. This unique distribution pattern of HBV/C subgenotypes is of interest; thirteen (C1, C2, C5, C6, C8-16) of the sixteen existing HBV/C subgenotypes prevail in Indonesia, with some confined to certain parts of the archipelago. This situation is in contrast with mainland Asia, where only two subgenotypes (C1 and C2) are observed.

HBV genetic diversity has been suggested to be associated with natural selection influenced by host ethnic-related genetic background [30], reflected by divergence of amino acid substitutions within certain regions of HBV structural proteins, particularly HBsAg and the core (HBcAg) antigens [31]. These two proteins are important because HBsAg contains T cell and B cell epitopes that define HBV variants [32–34], while HBcAg possesses immunologic targets of host immune response that determine the course of HBV infection [31,35]. Several Human Leukocyte Antigen (HLA)-restricted T cell epitopes within HBsAg and HBcAg have been proposed and different epitopes may present in consequence of the diverse distribution of HLA in populations in distinct geographical regions [36].

Studies on the association between genetic variation of HBV and the host have been reported [23,37,38]. The variation of HBV genetic characteristics has been extensively investigated for genotype B [23,39], but largely undefined for genotype C. Further, the knowledge on how the host-virus interaction shapes the molecular epidemiology pattern of HBV infection remains unclear. With ethnic diversity among the highest in the world, the Asia-Pacific region offers a unique host setting for HBV infection [40]; its coincidence with the highly diverse distribution of HBV/C subgenotypes has never been studied. We carried out this study to investigate the association between HBV/C molecular characteristics and its geographical distribution, by examining various subgenotypes of HBV/C isolates from the Asia and Pacific region, with further analysis on the immune epitope characteristics of the core and surface proteins.

## Materials and Methods

### HBV complete genome sequences and genetic relatedness analysis

Sixty-nine HBV complete genome sequences (Table 1) were retrieved from GenBank, including 62 isolates of the 16 existing HBV/C subgenotypes: 37 [C1 (3), C2 (1), C5 (3), C6 (12), C8 (4), C10 (1), C11 (2), C12 (4), C13 (3), C14 (2), C15 (1), and C16 (1)] from various geographical regions and ethnic populations of the Indonesian archipelago [19,23,27–29,39] and 25 [C1 (7), C2 (8), C3 (2), C4 (2), C5 (4), C7 (1), and C9 (1)] from other countries in Asia (Korea, China, Japan, Myanmar, Thailand, Vietnam, Malaysia, Philippines, and Timor Leste), the Pacific (Polynesia and New Caledonia), and Northern Australia, together with 7 isolates representing HBV/A (1), HBV/B (1), HBV/D (1), HBV/E (1), HBV/F (1), HBV/G (1), and HBV/H (1).

**Table 1:** HBV sequences used in this study.

No	Analysis method	Complete genome sequences^†^		Additional S gene sequences^†^		Additional C gene sequences^†^		Total sequences
		n	Source	n	Source	n	Source	used
1	Phylogenetic tree	62 HBV/C;	GenBank;					
	construction	7 various HBV genotypes;	GenBank;	-	-	-	-	69+1 outgroup
		1 WMHBV	GenBank					
2	Nucleotide divergence	57 HBV/C^‡^;	GenBank;	-	-	-	-	102
	analysis	45 additional HBV/C	GenBank					
3	Subgenotype distribution	62 HBV/C	GenBank	48 HBV/C Asia;	GenBank;			
				74 HBV/C Papua-Pacific;	GenBank;	-	-	271
				87 HBV/C Indonesia	This study			
4	Subtype distribution	62 HBV/C	GenBank	48 HBV/C Asia;	GenBank;			
				74 HBV/C Papua-Pacific;	GenBank;	-	-	271
				87 HBV/C Indonesia	This study			
5	Surface immune epitope							
	analysis							
	a. Amino acids s20-s180	62 HBV/C	GenBank	48 HBV/C Asia;	GenBank;	-	-	184
				74 HBV/C Papua-Pacific	GenBank			
	b. Amino acids s124-s148	62 HBV/C	GenBank	48 HBV/C Asia;	GenBank;			
				74 HBV/C Papua-Pacific;	GenBank;	-	-	271
				87 HBV/C Indonesia	This study			
6	Core immune epitope	62 HBV/C	GenBank	-	-	44 HBV/C Asia^§^;	GenBank;	143
	analysis^§^					37 HBV/C Papua-Pacific	GenBank	

^†^ Details of the GenBank Accession Numbers for all HBV sequences are provided in S1 Table.

^‡^ Five HBV/C sequences initially used in phylogenetic tree construction representing HBV/C7, C9, C10, C15, and C16 were not used in nucleotide divergence analysis because only single complete genome isolates were available for each of these subgenotypes.

^§^ Core immune epitope analysis used sequences that cover the C gene region. Compared to the sequences used in the surface immune epitope analysis, only 16 HBV/C Asia sequences were used again in the core immune epitope analysis, while all S gene sequences from HBV/C Papua Pacific and the 87 HBV/C Indonesia of this study did not qualify for the core immune epitope analysis.

The 69 HBV sequences were aligned using ClustalW software (http://www.ebi.ac.uk/ClustalW/) and confirmed by visual inspection. Phylogenetic tree was constructed by Monte Carlo Markov Chain (MCMC) method in Bayesian Inference software [41]. To have convergence data, analysis was run for ten million generations, and sampled once every 1,000 generations. The sumt and sump were run for 2,500 trees and the consensus tree was constructed based on 7,500 trees. HBV strain of woolly monkey hepatitis virus (AY226578) was used as outgroup.

To define the magnitude of inter-genotype and intra-genotype differences between HBV/C subgenotypes along with other HBV genotypes, pairwise analysis of nucleotide divergence was performed for 57 of the 62 HBV/C isolates. Pairwise distance calculation and Kimura2-parameter substitution model were used to analyze the genomic divergence. In this analysis, five subgenotypes (C7, C9, C10, C15, and C16) were not included because only single isolate was available for each. To increase data validity, 45 [C1 (17), C2 (25), C5 (1), C6 (2)] additional HBV/C complete sequences from the Asia and Pacific were searched from GenBank and analyzed together with the 57 HBV/C isolates, making a total of 102 complete genome sequences (Table 1).

### Additional sequences for HBV/C subgenotype distribution study and sample preparation

To obtain a better understanding of HBV/C subgenotype distribution in the Indonesian archipelago, surface (S) gene sequences were generated from 87 samples with known HBV subgenotypes as determined in our previous study [23]: [C1 (53), C2 (22), C5 (6) and C6 (6)]. The samples were collected on general hepatitis B screening of ethnically-defined, apparently-healthy populations from the islands of Sumatra (36), Kalimantan (1), Sulawesi (9), Flores (19), Sumba (1), Alor (3), Ternate-North Moluccas (5), Ambon-South Moluccas (7), and Papua of the Indonesian archipelago (6)—named Papua hereafter, as depicted in S1 Fig. Written informed consent was obtained from every participant recruited, and this study was approved by the Eijkman Institute Research Ethics Commissions (EIREC No. 23/2007).

HBV DNA was extracted from 140 μL serum sample using QIAamp viral DNA Mini Kit (Qiagen Inc., Chatsworth, CA) according to the manufacturer’s instruction. PCR amplification of part of the S gene region (226 bp) was carried out following a nested strategy using two oligonucleotide primer pairs: S2-1 (5’-CAAGGTATGTTGCCCGTTTG-3’, nt 455–474) and S1-2 (5’-CGAACCACTGAACAAATGGC-3’, nt 704–685) for the first round; S088 (5’-TGTTGCCCGTTTGTCCTCTA-3’, nt 462–471) and S2-2 (5’-GGCACTAGTAAACTGAGCCA-3’, nt 687–668) for the second round [10,42]. Denaturizing, annealing and extension were carried out at 94°C for 30 s, 55°C for 30 s and 72°C for 1 min, respectively, for both rounds of PCR (35 cycles for the first and 25 for the second round). Amplification products were directly sequenced using Big Dye Terminator Reaction kits with ABI 3130 XL genetic analyzer (ABI Perkin Elmer, Norwalk, CT, USA).

To put HBV/C subgenotype distribution in the context of its diversity in Asia-Pacific, additional S gene sequences of 48 HBV/C isolates from Asia and 74 from the Papua-Pacific [10 from PNG, 20 from Vanuatu, 20 from Tonga, 20 from Fiji, and 4 from Kiribati islands] were downloaded from GenBank. Thus, together with the 87 newly generated and the initial 62 complete genome HBV/C isolates, we examined 271 sequences to study the distribution pattern of HBV/C subgenotypes in the Asia and Papua-Pacific (Tables 1 and 2).

**Table 2:** Distribution of HBsAg subtypes and HBV genotypes/subgenotypes of 271 HBV/C isolates according to their country/geographical origins in East/Southeast Asia and Papua-Pacific.

Origins	N	Subtype	n	Genotype/subgenotype																
				C1	C2	C3	C4	C5	C6	C7	C8	C9	C10	C11	C12	C13	C14	C15	C16	C
China	16	*adrq+*	15	2	13															
		*adw2*	1		1															
Japan		*adrq+*	17		17															
	21	*adw2*	2		2															
		*ayr*	1		1															
		*adr_indet (A159/A177)*	1		1															
Korea	3	*adrq+*	2		2															
		*adr_indet (A159/A177)*	1		1															
Myanmar	8	*adrq+*	7	7																
		*adw2*	1	1																
Thailand	8	*adrq+*	7	7																
		*adr_indet (V159/V177)*	1	1																
Vietnam	6	*adrq+*	5	5																
		*ayr*	1	1																
Malaysia	1	*adrq+*	1	1																
Philippine	5	*adw2*	4					4												
		*adrq+*	1							1										
Indonesia		*adrq+*	78	38	19				9		4		1		4		2	1		
		*adw2*	17	5	3			9												
	124^#^	*ayr*	15	12										2					1	
		*ayw1*	2	1	1															
		*adr_indet (A159/A177)*	12						9							3				
Timor Leste	1	*adrq+*	1									1								
Australia	2	*ayw3*	2				2													
Polynesia	1	*adrq-*	1			1														
N. Caledonia	1	*adrq-*	1			1														
PNG	10	*adrq+*	8																	8
		*adr_indet (V159/V177)*	2																	2
Vanuatu		*adrq-*	15																	15
	20	*adrq+*	1																	1
		*adr_indet (V159/V177)*	3																	3
		*ayr*	1																	1
Fiji		*adrq-*	13																	13
	20	*adr_indet (V159/V177)*	6																	6
		*ayw1*	1																	1
Tonga	20	*adrq-*	19																	19
		*adr_indet (V159/V177)*	1																	1
Kiribati	4	*adrq-*	3																	3
		*adrq+*	1																	1
**Total**			**271**	**81**	**61**	**2**	**2**	**13**	**18**	**1**	**4**	**1**	**1**	**2**	**4**	**3**	**2**	**1**	**1**	**74**

^# including 37 published complete genome sequences and 87 newly generated in this study; N. Caledonia: New Caledonia; PNG: Papua New Guinea.^

### HBsAg subtype determination of HBV/C strains

Deduced amino acid sequences of the S gene from the 271 HBV/C isolates were aligned using BioEdit package version 7.0 software. Amino acid variations that determine HBsAg subtypes (*adw*, *adr*, *ayw*, and *ayr*) were identified based on the common antigenic determinant *‘a’* at amino acids 124–147 (S1 and S2 Figs), and two pairs of mutually exclusive determinants, *d*/*y* and *w*/*r*, at amino acids s122 and s160, respectively [43]. Further specification into nine subtypes (*ayw*1, *ayw*2, *ayw*3, *ayw*4, *ayr*, *adw*2, *adw*3, *adw*4, *adrq*+ and *adrq*-) based on previous reports was also accomplished [13,14].

### Examination of HBV/C surface and core immune epitopes

Because of the limited availability of HBV/C sequences, particularly from the Papua-Pacific islands, examination of known immune epitopes within HBV/C surface and core regions could not be totally performed on full-length genome sequences. It was only done on either surface or core sequences available in public databases [30], or from additional isolates generated in this study (Table 1). Examination for known recognition sites encompassing residues s20-s180 of HBsAg was accomplished in 184 of the 271 isolates, while the shorter sequence of the remaining 87 isolates generated in this study allowed only for B cell epitope analysis within residues s124–s148 (S1 and S2 Figs). Analysis of core immune epitopes was done for a total of 143 isolates representing various geographical regions in Asia and Pacific as shown in S3 Fig. The 87 sequences from this study were not included in this analysis due to insufficient volume of the repository specimens. The analysis was performed by comparing the cytotoxic T lymphocyte (CTL) recognition sites, as well as T helper and B cell immune epitopes of HBV/C isolates from East and Southeast Asia (Japan, Korea, China, Hongkong, Vietnam, Myanmar, Thailand, Malaysia, and Indonesia), and Papua-Pacific region (Papua New Guinea, Polynesia, New Caledonia, as well as Vanuatu, Fiji, and Tonga islands) with HBV/C1 isolate AF 473543 from China used as the reference (S3 Fig). To assess the significance of epitope variations among different isolate groups, we performed statistical analysis by Pearson’s chi-square test (significant p-value <0.05) using SPSS v.20 software.

## Results

### Phylogenetic analysis of HBV complete genome sequences and nucleotide divergence of HBV/C strains

**Fig 1:**
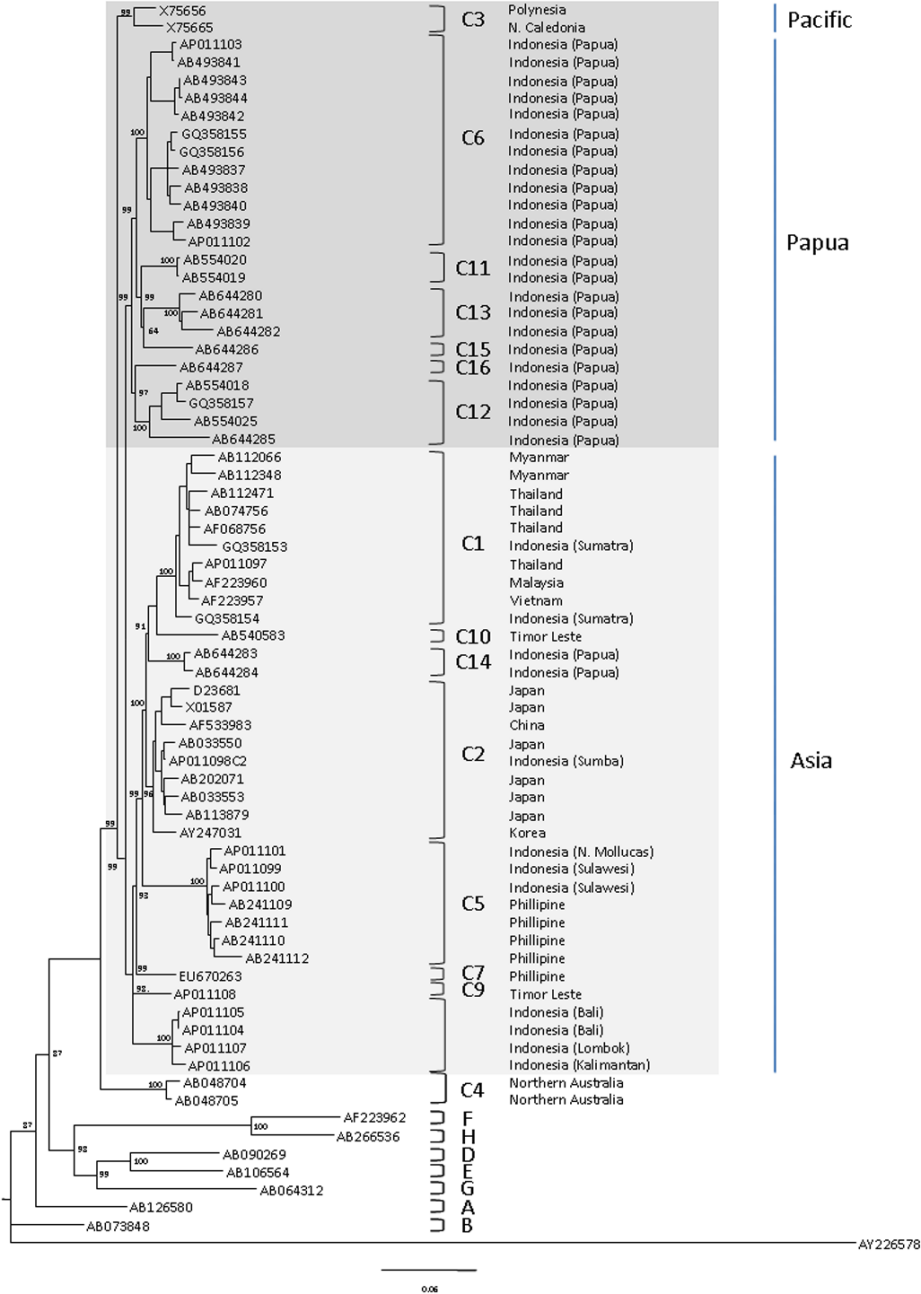
Phylogenetic tree of HBV/C isolates from different countries in East and Southeast Asia, and Papua-Pacific. A Bayesian phylogenetic tree analysis based on complete genome sequences showed that isolates from various subgenotypes (C1-C16) are clearly grouped into two major clusters, consistent with their geographical origins. Seven HBV/C subgenotypes (C1, C2, C5, C7, C8, C9, and C10) from East and Southeast Asia, and one (C14) from Papua (*light highlight*) were well-separated from those six subgenotypes (C6, C11, C12, C13, C15, and C16) from Papua, and from one subgenotype (C3) from Pacific, the more east region of the Papua (*dark highlight*). Although the root of subgenotype C3 phylogenetically is distanced from the subgenotypes of Papua, the isolate geographic origin, the immune epitope characteristics of surface and core proteins, and the HBsAg subtype gradient distribution showed these HBV/C3 isolates to be close to Papua subgenotypes. Therefore, the Papua and the Pacific subgenotypes are classified together into Papua-Pacific type. The diversification of the Asian type from the Papua-Pacific type started from Papua of Indonesia to the east. The other subgenotype, HBV/C4, was distanced from other subgenotypes. In this analysis, one strain (GQ358157) from Papua reported as C6 in our previous study [23] grouped into C12. We redefine this strain as a member of HBV/C12.

Phylogenetic analysis based on 69 HBV complete sequences retrieved from GenBank confirmed the clustering of eight HBV genotypes and their subgenotypes as shown in Fig 1. Interestingly, of 62 HBV/C isolates, two major clusters were observed: one of 35 isolates [C1 (10), C2 (9), C5 (7), C7 (1), C8 (4), C9 (1), C10 (1), and C14 (2)], and the other of 23 isolates [C6 (12), C11 (2), C12 (4), C13 (3), C15 (1), and C16 (1)]. The first cluster associated mainly with Southeast and East Asian countries, while the second cluster with those of Papua. Two isolates of C3 which are specific to Pacific region has distinct root with those of Asia and Papua clusters, and the remaining two C4 isolates of Northern Australia belonged to the more distinct lineage that was more distant compared to the two clusters and the Pacific isolates.

Over the complete genome of 102 HBV/C isolates, the nucleotide divergence between subgenotypes was higher than 4% (Table 3), with the exception of C2 to C14 (3.91%). The genetic divergence among HBV/C subgenotypes specific for Indonesia (C6, C8, C11, C12, C13, and C14) was as high as 5.92% for C12 to C13, while C6 to C11 had the lowest genetic divergence (4.02%). HBV/C1 and C2, which are specific for the Asian mainland, showed low genetic divergence (4.37%). Among all HBV/C isolates, C12 had the highest intra-subgenotype nucleotide divergence (3.25 ± 1.58%). The most deviating cluster, HBV/C4, had the highest evolution distinction to C1, C2, C3, C5, C6, C8, C11, C12, C13, and C14, supported by 7.16%, 6.36%, 6.01%, 7.71%, 6.57%, 6.26%, 6.19%, 6.69%, 7.44%, and 6.77% nucleotide divergence, respectively.

**Table 3:** Mean percentage nucleotide divergence of the complete genome between HBV/C subgenotypes. The total number of HBV/C isolates examined for each subgenotype is shown in bracket. Other existing subgenotypes (C7, C9, C10, C15, and C16) were not included in the genetic distance calculation since only single isolate was available for each subgenotype. Intrasubgenotype divergences are shown in bold.

	C1 (27)	C2 (34)	C3 (2)	C4 (2)	C5 (8)	C6 (14)	C8 (4)	C11 (2)	C12 (4)	C13 (3)	C14 (2)
C1	**2.68 ± 0.65**										
C2	4.37 **±** 0.52	**2.52 ± 0.62**									
C3	4.86 **±** 0.48	4.41 **±** 0.34	**2.71 ± 1.91**								
C4	7.16 **±** 0.41	6.36 **±** 0.35	6.01 **±** 0.24	**0.91 ± 0.64**							
C5	5.65 **±** 0.39	5.27 **±** 0.40	5.92 **±** 0.32	7.71 **±** 0.30	**1.55 ± 0.48**						
C6	5.78 **±** 0.44	4.66 **±** 0.33	4.49 **±** 0.27	6.57 **±** 0.25	6.36 **±** 0.28	**2.53 ± 0.94**					
C8	5.30 **±** 0.38	4.28 **±** 0.28	4.71 **±** 0.24	6.26 **±** 0.22	5.59 **±** 0.23	4.94 **±** 0.23	**0.77 ± 0.38**				
C11	5.64 **±** 0.43	4.71 **±** 0.24	4.49 **±** 0.20	6.19 **±** 0.22	6.44 **±** 0.19	4.02 **±** 0.17	4.95 **±** 0.14	**0.19 ± 0.13**			
C12	6.05 **±** 0.51	5.07 **±** 0.51	5.03 **±** 0.50	6.69 **±** 0.42	6.62 **±** 0.39	4.98 **±** 0.56	5.28 **±** 0.41	4.91 **±** 0.56	**3.25 ± 1.58**		
C13	6.19 **±** 0.41	5.64 **±** 0.36	5.57 **±** 0.28	7.44 **±** 0.33	7.29 **±** 0.29	5.01 **±** 0.41	5.75 **±** 0.29	4.72 **±**0.21	5.92 **±** 0.48	**2.26 ± 0.46**	
C14	4.61 **±** 0.42	3.91 **±** 0.31	4.83 **±** 0.17	6.77 **±** 0.16	5.78 **±** 0.29	5.03 **±** 0.11	4.82 **±** 0.09	5.25 **±** 0.06	5.36 **±** 0.53	5.64 **±** 0.25	**0.75 ± 0.53**

### Variation of amino acids within immune epitopes of HBV/C isolates

**Fig 2:**
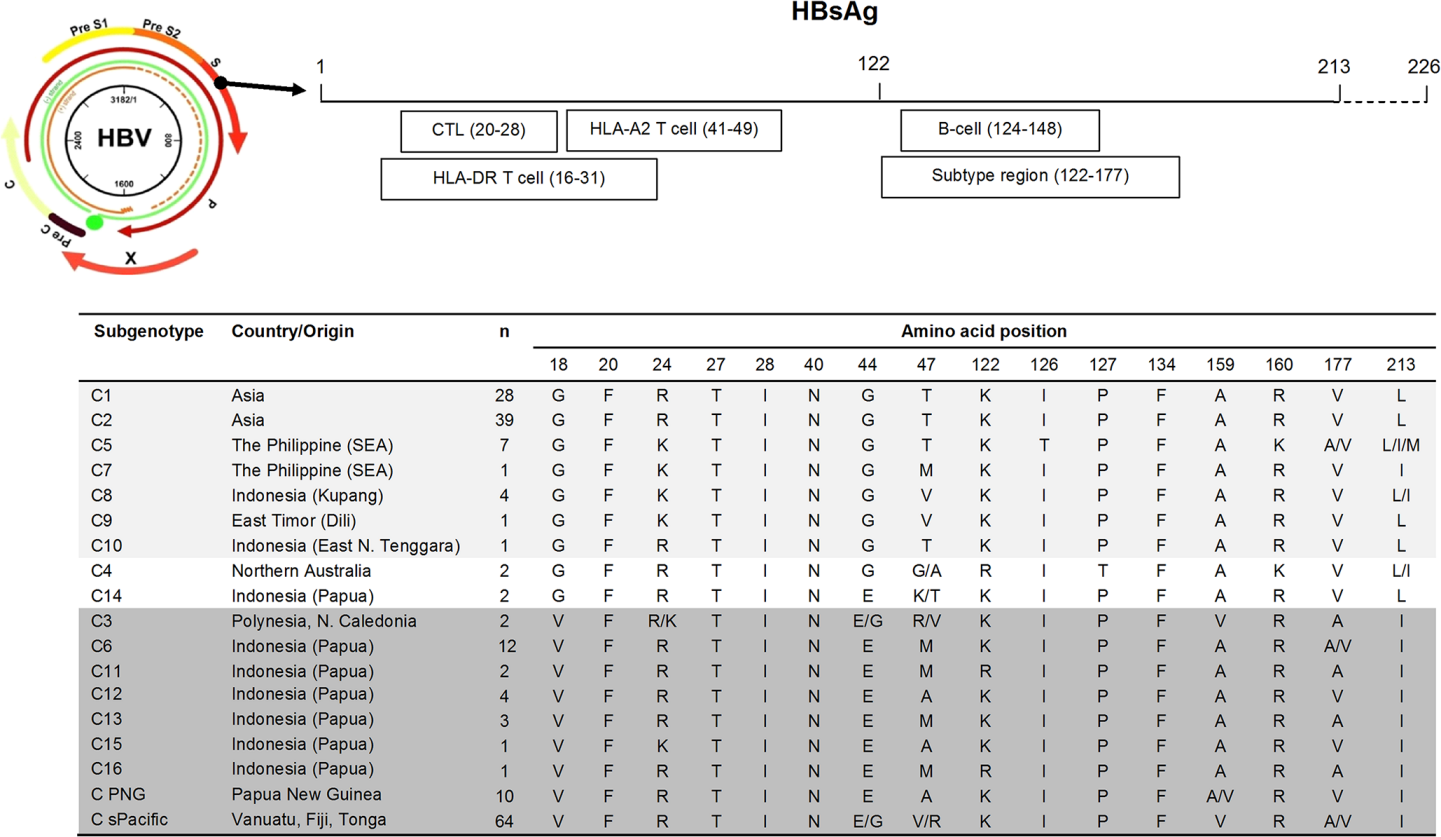
HBsAg amino acid motifs in B and T-cell epitopes of Asia and Papua-Pacific HBV/C isolates. For the sake of clarity, this figure was not drawn to scale. Using 184 isolates, analysis of HBV/C immune epitopes within HBsAg identified two frequently observed patterns of amino acid variations corresponding to the geographical origins of the isolates. The first pattern of seven subgenotypes represented HBV/C subgenotypes from Asia (*light highlight*), while the second pattern of seven subgenotypes and unclustered subgenotypes from PNG and Pacific (*dark highlight*) was from Papua and Pacific. Two subgenotypes, HBV/C4 and C14, did not belong to either Asia or Papua-Pacific clusters. We also identified sG18 and4 as the critical points distinguishing the Asian from the Papua-Pacific type (p-values <0.001; data not shown).

All the 87 S gene sequences generated in this study have been deposited in GenBank database [Accession numbers JQ740646-JQ740732]. Inspection of HBsAg HLA class-I-restricted CTL epitopes (residues s20–s28: FLLTRILTI) [44] from 184 HBV/C sequences showed an sR24K substitution in HBV/C3 isolate of New Caledonia, all HBV/C5 and C7 of the Philippines, as well as C5, C8, C9, and C15 of Indonesia (Fig 2). Substitutions were also observed at positions s44 and s47 located within a HLA class-I-restricted T cell epitope (residues s41–s49) [34]. At position s44, an sG44E substitution was identified in all HBV/C6, C11, C12, C13, C14, C15, C16, and unclassified HBV/C isolates from PNG and Tonga, with the majority of isolates from Fiji (18, 90%), Kiribati (3, 75%), and Vanuatu (8, 40%) also showed this pattern (S2 Fig). High amino acid variability was found at residue s47, particularly in Papua-Pacific HBV/C isolates (Figs 2 and S2). In the HBsAg B cell epitopes of the ‘*a’* determinant (residues s124–s148) [45,46], an sI126T substitution was only unanimously detected in HBV/C5 isolates, while sP127T was identified in all HBV/C4 and 2 (10%) HBV/C isolates from Vanuatu (Figs 2 and S2). Within a HLA class-II-restricted T cell epitope (residues s16-s31) [47,48], a sG18V switching was observed in all HBV/C6, C11, C12, C13, C15, C16, and majority of HBV/C isolates from PNG and from Pacific, but not in the other C subgenotypes. Furthermore, at residue s213, a substitution from L to I was detected in all isolates of HBV/C3, C6, C7, C9, C11, C12, C13, C15, C16, and C from PNG and from Pacific. Notably, most HBV/C6, C11, C12, C13, C15, and C16 isolates shared the same amino acid variation motifs with HBV/C isolates from Pacific with sV18, sE44, and sI213, while isolates of the other HBV/C subgenotypes had sG18, sG44, and sL213 motif. When these amino acid patterns were analyzed using Pearson’s chi-squared test, the frequency distributions were found to be statistically significant in distinguishing isolates from Asia and from Papua-Pacific (p-values <0.001 for all three patterns). In comparison, the most distanced subgenotype, HBV/C4, showed more substitutions: sG56, sT68, sS113, sT114, sT127, sA184, and sI198 (not shown).

**Fig 3:**
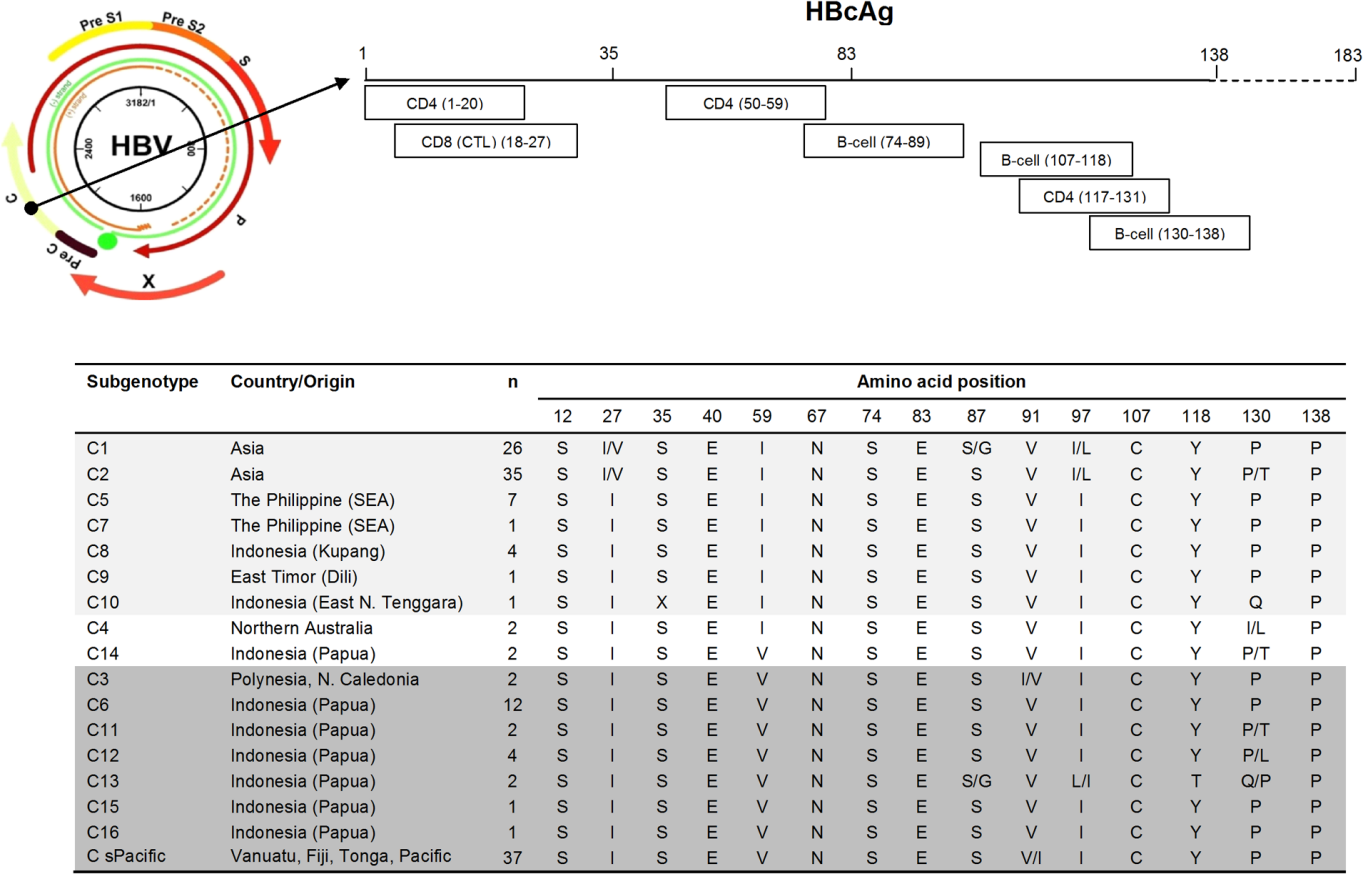
HBcAg amino acid motifs in B and T-cell epitopes of Asia and Papua-Pacific HBV/C isolates. For the sake of clarity, this figure was not drawn to scale. Among 15 amino acid positions examined within HBcAg immune epitopes of 143 isolates, we identified I/V at position c59 as the essential variation that classified HBV/C subgenotypes into two major clusters, the Asian and the Papua-Pacific (p-value <0.001; data not shown). HBV/C4 and C14 showed similar variation in most amino acids examined, with C4 and C14 having cI59 and cV59, respectively.

Result of the analysis of 143 amino acid sequences of HBcAg for known T helper, CTL, and B cell recognition sites is shown in Figs 3 and S3. In the T helper epitopes (residues c1-c20, c50-c69, and c117-c131), of the 77 HBV/C isolates from East and Southeast Asia, 12 isolates had variations within residues c1-c20 with cV13A as the most frequent substitution. In contrast, only 2 isolates from Papua and one isolate from Fiji of Papua-Pacific had amino acid substitutions [cI3V, cL16I, and cS12P/cE14A, respectively] (S3 Fig). Interestingly, within residues c50–c69, a single substitution—cI59V—distinguished the East and Southeast Asia isolates (HBV/C1, C2, C5, C7, C8, C9, and C10) from those of the Papua-Pacific (HBV/C3, C6, C11, C12, C13, C15, C16, and C Pacific). Using Pearson’s chi-squared test analysis, the p-value of this amino acid pattern distribution was found to be significant (p-value <0.001). Notably, HBV/C14 that was phylogenetically grouped into the East and Southeast Asian cluster (Fig 1) had cV59 that marked the Papua-Pacific strains (Figs 3 and S3). In residues c117–c131, 14 of 77 isolates from East and Southeast Asia and 6 of 64 isolates from Papua-Pacific had cP130T/L/Q/A substitution. The two most distinct HBV/C4 isolates from the Australian Aboriginals showed cI59 and cL/I130 with no variation within residues c1-c20.

For HLA class-I-restricted epitopes in HBcAg (residues c18–c27, c84–c101, and c141–c151) [37,49,50], the following observations were seen: (i) most HBV/C isolates from Asia and Papua-Pacific had cI27 within residues c18–c27 (FLPSDFFPSI), except for 5 isolates from Asia (Japan, China, Hongkong, Myanmar, and Vietnam) and 1 isolate from Papua that had cV27; (ii) various amino acid substitutions were observed in residues c84–c101, of which cV91 was consistently identified in isolates from East and Southeast Asia (C1, C2, C5, C7, C8, C9, and C10), Papua (C6, C11, C12, C15, and C16), and most of C13 isolates and those from Vanuatu of Pacific. However, HBV/C isolates from Polynesia (C3) and the majority of those from Fiji, Tonga, and Pacific had cI91. The most divergent subgenotype, HBV/C4, had cV91. Examination of HBcAg B-cell epitopes (residues c74–c89, c107–c118, c130–c138, and c148–c160) [31] showed no specific variation in all HBV/C isolates.

### Distribution of HBsAg subtypes

HBsAg subtypes of the 271 HBV/C isolates (164 of the East and Southeast Asia, 105 of the Papua-Pacific, and 2 of North Australia) were identified based on the translated amino acid variations as described in previous studies [13,14] (Table 2), including the combination of amino acids at positions s159 (A/V) and s177 (V/A) that specifies the subtype *adr* into *q+* and *q-* patterns [51]. A clear signature of HBsAg *q* subdeterminant variability that separated the HBV/C isolates from Asia and from Papua-Pacific was observed.

**Fig 4:**
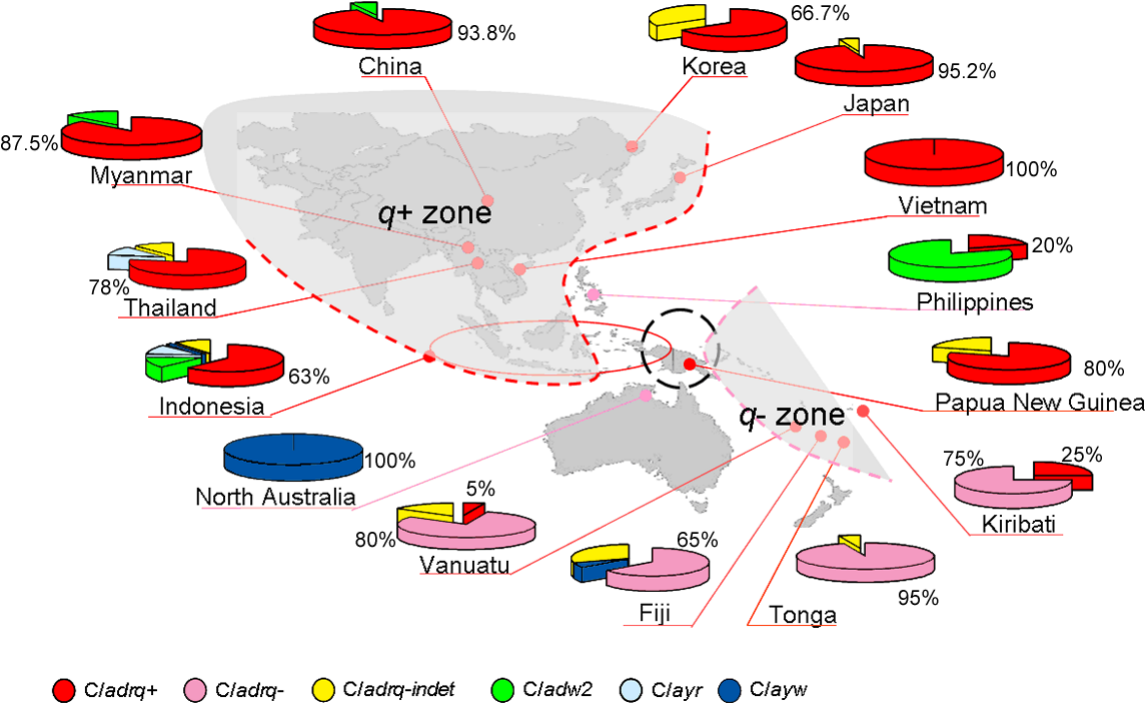
Distribution of HBV/C subtypes in the East and Southeast Asia and the Papua-Pacific. This study identified a west-to-east gradient in the distribution of HBsAg subtypes with *adrq+* (*red*) prominent in East-Southeast Asia and *adrq-* (*pink*) in the Pacific region (Vanuatu, Fiji, Tonga, and Kiribati). Interestingly, together with *adrq+*, *adrq-*indeterminate sA159/sA177 and a new pattern of *adrq-*indeterminate sV159/sV177 identified in this study were found in Papua and PNG, respectively, suggesting that the molecular admixture of HBV/C, particularly for subtype evolution, occurred in Papua and PNG with both *adrq-*indeterminate forms (*yellow*) as the transitional patterns.

The distribution of the HBsAg subtypes with respect to their countries/geographical origins is illustrated in Fig 4. Of 81 HBV/C1 isolates (Thailand 8, Vietnam 6, Myanmar 8, Malaysia 1, Indonesia 56, and China 2), the majority (60; 74.1%) could be classified into *adrq+* subtype, while 13 (16.1%), 6 (7.4%), and 1 (1.2%) belonged to *ayr*, *adw2* and *ayw1* subtypes, respectively. Interestingly, 1 (1.2%) strain from Thailand had Valine at both s159 and s177 residues. Since the unique sV159/sV177 combination has not been reported, we provisionally designate this pattern as another form of *adrq-*indeterminate, in addition to the previously reported *adrq-*indeterminate sA159/sA177 combination [19]. Likewise, of 61 HBV/C2 isolates (China 14, Japan 21, Korea 3, and Indonesia 23), most (51; 83.6%) could be classified into *adrq+*, while the rest belonged to *adw2* (6; 9.8%), *ayw1* (1; 1.6%), *ayr* (1; 1.6%), and *adrq-*indeterminate sA159/sA177 (2; 3.3%). All 13 HBV/C5 isolates from the Philippines and Indonesia belonged to *adw2* subtype, while isolates HBV/C7 from the Philippines, C9 from Timor Leste, and C8, C10 and C14 from Indonesia were of *adrq+* subtype.

Of 18 HBV/C6 isolates from Papua, 9 belonged to *adrq+* and 9 to *adrq-*indeterminate sA159/sA177 subtypes, whereas 2 C11, 4 C12, 3 C13, 1 C15, and 1 C16 isolates were of *ayr*, *adrq+*, *adrq-*indeterminate sA159/sA177, *adrq+*, and *ayr* subtypes, respectively. Of 10 HBV/C isolates from PNG, 8 had *adrq+* and 2 a*drq-*indeterminate sV159/sV177 subtypes. Further, among 64 HBV/C isolates from Pacific region (Vanuatu, Fiji, Kiribati, and Tonga), the majority of isolates (50; 78.1%) had *adrq-* subtype, while 10 belonged to *adrq-*indeterminate sV159/sV177, 2 to *adrq+*, 1 to *ayr*, and 1 to *ayw1* subtypes. Two HBV/C3 isolates derived from the Pacific region had *adrq*- subtype. Two HBV/C4 isolates from Australian Aboriginals were distantly related to both the Asian and the Pacific groups, showing a pattern typical for *ayw3* subtype.

## Discussion

### Diversity of HBV/C strains in Indonesia with two major types: Asian and Papua-Pacific

Positioned in the middle of Asia-Pacific, Indonesia with around 500 ethnic populations dispersed in thousands of island, has a unique distribution of HBV genotypes and subgenotypes [39,52]. Further, the presence of thirteen HBV/C subgenotypes (C1, C2, C5, C6, C8-C16) in Indonesia is remarkable as to date no other region has been reported to have such a high variety of HBV/C isolates. This fact demonstrates that Indonesia has a much greater HBV/C genome diversity than other regions in Asia, in contrast to East Asian countries that have a more homogenous distribution of HBV/C subgenotypes. This varying distribution was also seen across the Indonesian archipelago, from two (C1 and C2) in the west to more heterogeneous (C5, C6, C8-C16) subgenotypes in the east. Phylogenetic analysis of HBV/C complete genome clearly demonstrated the separation of HBV/C subgenotypes of Asian (East and Southeast Asian) countries (C1, C2, C5, C7, C8, C9, C10, and C14) from those of Papua (C6, C11, C12, C13, C15, and C16) and Pacific region (C3) as seen in Fig 1. Of interest is the C3 subgenotype: by phylogenetic tree, the C3 is distanced from the Asia and Papua subgenotypes; however, by examining immune epitope characteristics of surface and core proteins, markedly the C3 subgenotype belonged together with Papua type. Further, with regard to the geographical origins and the consistency of HBsAg subtype gradient distribution, we, therefore, classified these Papua and Pacific subgenotypes into Papua-Pacific type. Thus, this study reveals that 15 of HBV/C subgenotypes clustered into two major types, the Asian and the Papua-Pacific, whereas one subgenotype (C4) was confined to Northern Australia and had distinct genome characteristics (Table 3).

The segregation of HBV/C subgenotypes into the Asian and Papua-Pacific groups is in line with the host ethno-geographical and linguistic distribution in Asia-Pacific. It has been recognized that genetic ancestry is strongly correlated with linguistic affiliations as well as geography in this region [53]. Consistent with previous reports, two subgenotypes (C1 and C2) were prominent in Southeast Asia and East Asia, respectively, with homogenous distribution [15,22,54]. In contrast, the other six subgenotypes (C5, C7, C8, C9, C10, and C14) were observed only in Southeast Asia with heterogeneous distribution.

Result of the examination of variants within the CTL immune recognition sites of HBV/C core region is in agreement with the genetic separation, showing a clear division of Asian and Papua-Pacific patterns. Significantly, we discovered a critical polymorphism c59I/V within the 183 amino acids of HBcAg distinguishing HBV/C into the Asian and Papua-Pacific types, with the exception of C14 that has Papua-Pacific c59V characteristics (Fig 3). This c59I/V polymorphism warrants further study since it is located in the highly immunogenic T helper epitopes [33,35].

The amino acid sequence of the core region as the important target for immune-mediated viral clearance by CTL, T helper, and B cells’ responses is relatively conserved [15,55]. However, variations within the core region have been observed and associated with its function in induction of host immune response [31,38,56]. In this study, by using HBcAg specific motifs recognized in the Asian and Papua-Pacific populations [3,57], we discovered that the isolates from East and Southeast Asia had more amino acid substitutions compared to those from Papua-Pacific (Fig 3). In keeping with the reported low divergence of HBV strains in Papua-Pacific [57], the higher conservation of HBcAg could be attributable to the more homogeneous immunity exerted by hosts of genetically less diverse populations in this geographical region [58]. This specific HBV core amino acid variation could be a consequence of host-virus interaction that shape the HBV-specific T-cell repertoire, and partly influenced by parallel evolution of geographically separated HBV lineages in the Asia and Papua-Pacific [57,59]. Population-based studies in both regions are needed to understand the role of host factors in the selection of HBV strains as well as its clinical and public health implications.

The characteristics of immune epitopes of surface protein further support the segregation of HBV/C subgenotypes into the Asian and Papua-Pacific groups. We discovered three unique substitutions—sG18V, sG44E, and sL213I—that separate the two groups (Fig 2). Interestingly, two of these substitutions are located within important immune epitopes: sG18V in the HLA class-II-restricted T-cell epitope (residues s16-s31) and sG44E in the HLA class-I-restricted T-cell epitope (residues s41-s49). The HLA class-II-restricted epitope (residues s16-s31) has been shown to have variable capacity in eliciting antibody response to HBsAg vaccination [47], while the HLA class-I-restricted epitope (residues s41-s49) is located in the hotspot mutational domain of HBsAg (residues s28-s51). It was hypothesized that this domain could contribute to the protective cellular immunity in natural infection with HBV, and mutations within this domain could predispose the hosts to chronic infection [34]. Future functional studies on these mutations would lead to a better understanding of the complex virus-host interactions.

Some anomalies were also observed in this study. Two HBV/C14 isolates (AB644283 and AB644284) recently found in Papua clustered together with strains from Asia (Fig 1). These isolates have s44E of the surface protein and c59V of the core protein characteristics of the Papua-Pacific type (Figs 2 and 3), but they have s18G of the surface protein that marks the Asian type (Fig 2). More isolates of this subgenotype are expected to explain this phenomenon, since the two isolates were derived from hosts with different ethnic backgrounds, i.e. Austronesian and non-Austronesian [29]. The two C4 isolates from Aboriginal population of Australia showed a distinct cluster unclassifiable into either the Asian or Papua-Pacific types, suggesting that this subgenotype might have a molecular evolution different from the other C subgenotypes.

All the analyses above indicate the presence of two types of HBV/C subgenotypes: the Asian and Papua-Pacific, based on the genetic characteristics of complete genome and the immune recognition sites within core and surface proteins. This finding is consistent with the host ethnical and geographical association as proved by the linguistic evidence: HBV/C of Asian type with the Austronesian speaking populations in the East and Southeast Asia, and HBV/C of Papua-Pacific type with the Papuan speaking populations in the Papua-Pacific region [60,61]. Taken together, this finding could suggest that the distribution of HBV/C isolates follows linearly the prehistorical human dispersal, in agreement with our previous report on HBV/B subgenotype distribution in Indonesia [23].

### A west-to-east gradient of HBsAg subtype distribution with Papua and Papua New Guinea as the HBV/C admixture region

Among 162 HBV/C isolates (HBV/C1, C2, C5, C7, C8, C9, C10) from the East and Southeast Asian countries (China, Japan, Korea, Myanmar, Vietnam, Thailand, Philippines, Malaysia, Indonesia, and Timor Leste), the majority were *adrq+* (72.8%), followed by *adw2* (15.4%), *ayr* (8.6%), and *ayw1* (1.3%); while *adrq-*indeterminate sA159/sA177 and a novel *adrq-*indeterminate sV159/sV177 accounted for 1.3% and 0.6%, respectively (Table 2). A closer look to the Indonesian archipelago revealed specific pattern of HBsAg subtype distribution, with predominance of *adrq+* spanning from the western part to Nusa Tenggara islands in the east. In contrast to East and Southeast Asia, among 64 HBV/C isolates from the Pacific region (Vanuatu, Fiji, Tonga, and Kiribati islands), *adrq*- was the most prevalent (78.1%), followed by *adrq-*indeterminate sV159/sV177 (15.6%), *adrq*+ (3.1%), *ayr* (1.6%), and *ayw1* (1.6%) (Table 2). These findings show a west-to-east gradient of HBsAg subtype distribution of HBV/C with *adrq*+ prominent in Asia and *adrq*- in the Pacific.

In Papua and PNG, the regions between Southeast Asia and the Pacific, both *adrq-*indeterminate forms prevail in addition to *adrq+* and *adrq-*: sA159/sA177 in Papua and sV159/sV177 in PNG. Subtype *adrq-*indeterminate sA159/sA177 represents 38.7% of HBV/C in Papua, while subtype *adrq-*indeterminate sV159/sV177 accounts for 20.0% of HBV/C in PNG. This specific distribution could show that Papua and PNG are the regions where the switching from *adrq*+ (72.8% in Asia) to *adrq*- (78.1% in Pacific) occurred, characterized by the presence of the two *adrq-*indeterminate as intermediate forms. The importance of these geographical regions as the transitional zone of past migratory events from Asia into Pacific [62], followed by long standing isolation of these populations, could be the background of this HBV/C genetic segregation in Papua and PNG.

## Conclusions

Our data indicated that HBV/C isolates can be classified into two types, the Asian and the Papua-Pacific, based on the virus genome diversity, immune epitope characteristics, and geographical distribution, with molecular evolutionary admixture occurred in Papua and PNG. More HBV/C isolates could be expected from these regions, since the chance of having HBV/C genetic admixture is greater there. Further investigation covering the scientific, clinical and public health aspects of the two types of HBV/C subgenotypes, together with other genotypes and subgenotypes prevailing in this region, need to be undertaken. This information would provide insights into the development of disease management strategy and the design of diagnostic tools and vaccine for HBV infection in such a genetically diverse host population.

## Supporting Information

S1 Fig The amino acid sequences of the 87 HBV/C isolates generated in this study. A total of 87 HBV/C isolates of various HBV/C subgenotypes [Accession numbers JQ740646-JQ740732] were collected from ethnically-defined hosts from various geographical regions of the Indonesian archipelago. Analysis was performed for variations of the amino acids of the surface protein (HBsAg) from residues s104-s178 including the subtype-determining amino acids.
(TIF)

S2 Fig The immune epitopes in the surface protein (HBsAg) of 184 HBV/C isolates. In this study, 184 isolates of various HBV/C subgenotypes were examined for immune epitopes within the surface protein (HBsAg) including the subtype-determining amino acids. The isolates were retrieved from GenBank following their origins from various geographical regions in the East and Southeast Asia and the Papua-Pacific. Variations sG18V, sG44E, and sL213I were detected in isolates from Papua-Pacific. These variations grouped all isolates into two clusters, the Asia and the Papua-Pacific (p-values <0.001; data not shown).
(TIF)

S3 Fig The immune epitopes in the core protein (HBcAg) of 143 HBV/C isolates. In this study, 143 isolates of various HBV/C subgenotypes were examined for immune epitopes within the core protein (HBcAg). The isolates were retrieved from GenBank following their origins from various geographical regions in the East and Southeast Asia and the Papua-Pacific. In general, compared with HBV/C isolates from Papua-Pacific, C isolates derived from Asia showed higher amino acid variation. A single amino acid variation—cI59V—markedly demonstrated the clustering of isolates from Asia and Papua-Pacific (p-value <0.001; data not shown).
(TIF)

S1 Table List of Accession Numbers of HBV sequences used in this study. (DOC)
