# Supplementary figures and images for: Genogeography and Immune Epitope Characteristics of Hepatitis B Virus Genotype C Reveals Two Distinct Types: Asian and Papua-Pacific
Source: PLoS One. 2015 Jul 10;10(7):e0132533. doi: 10.1371/journal.pone.0132533 (PMC4498642; doi:10.1371/journal.pone.0132533)

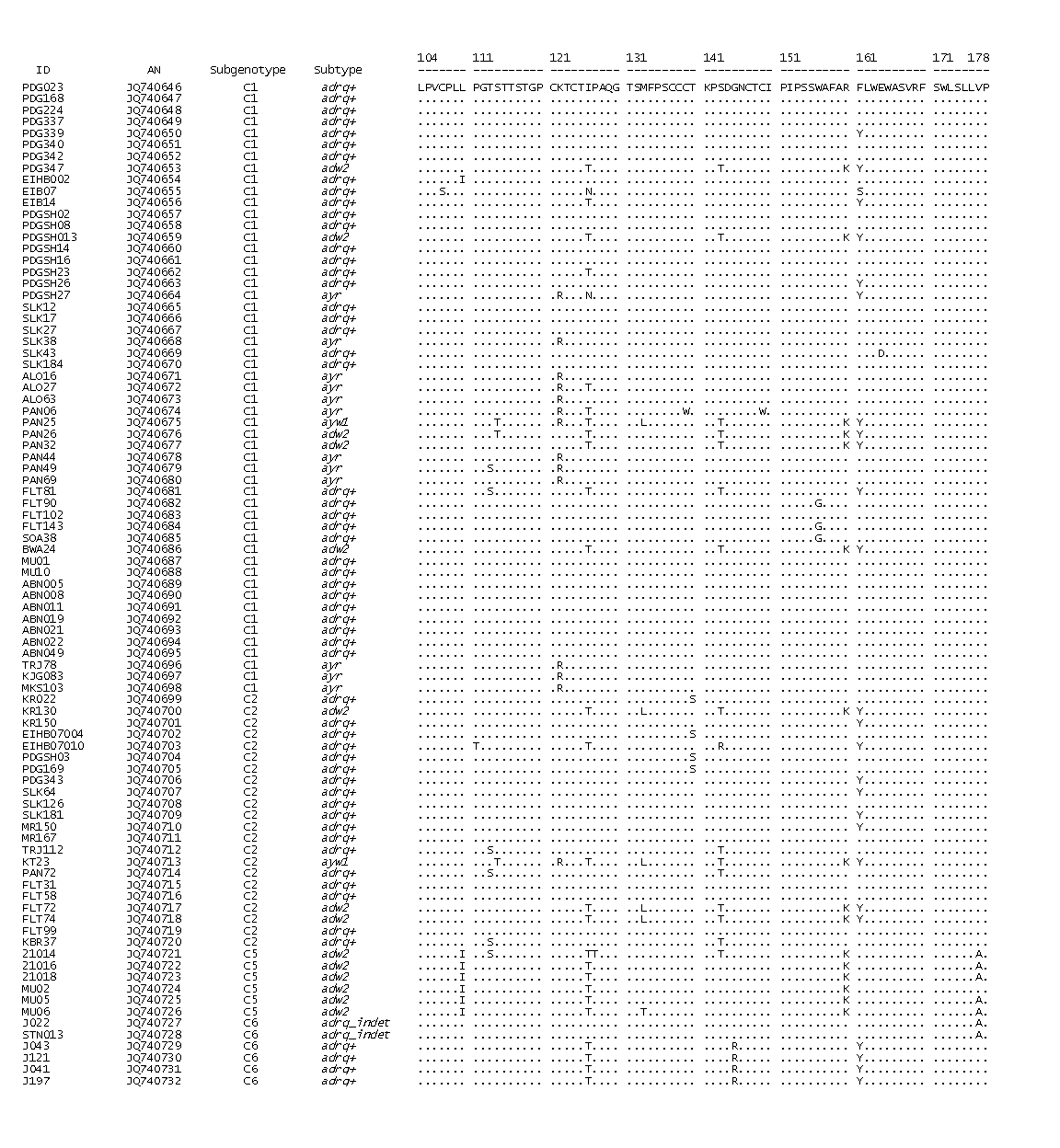

Supplement: S1 Fig — A total of 87 HBV/C isolates of various HBV/C subgenotypes [Accession numbers JQ740646-JQ740732] were collected from ethnically-defined hosts from various geographical regions of the Indonesian archipelago. Analysis was performed for variations of the amino acids of the surface protein (HBsAg) from residues s104-s178 including the subtype-determining amino acids. (TIF) [file pone.0132533.s001.tif]

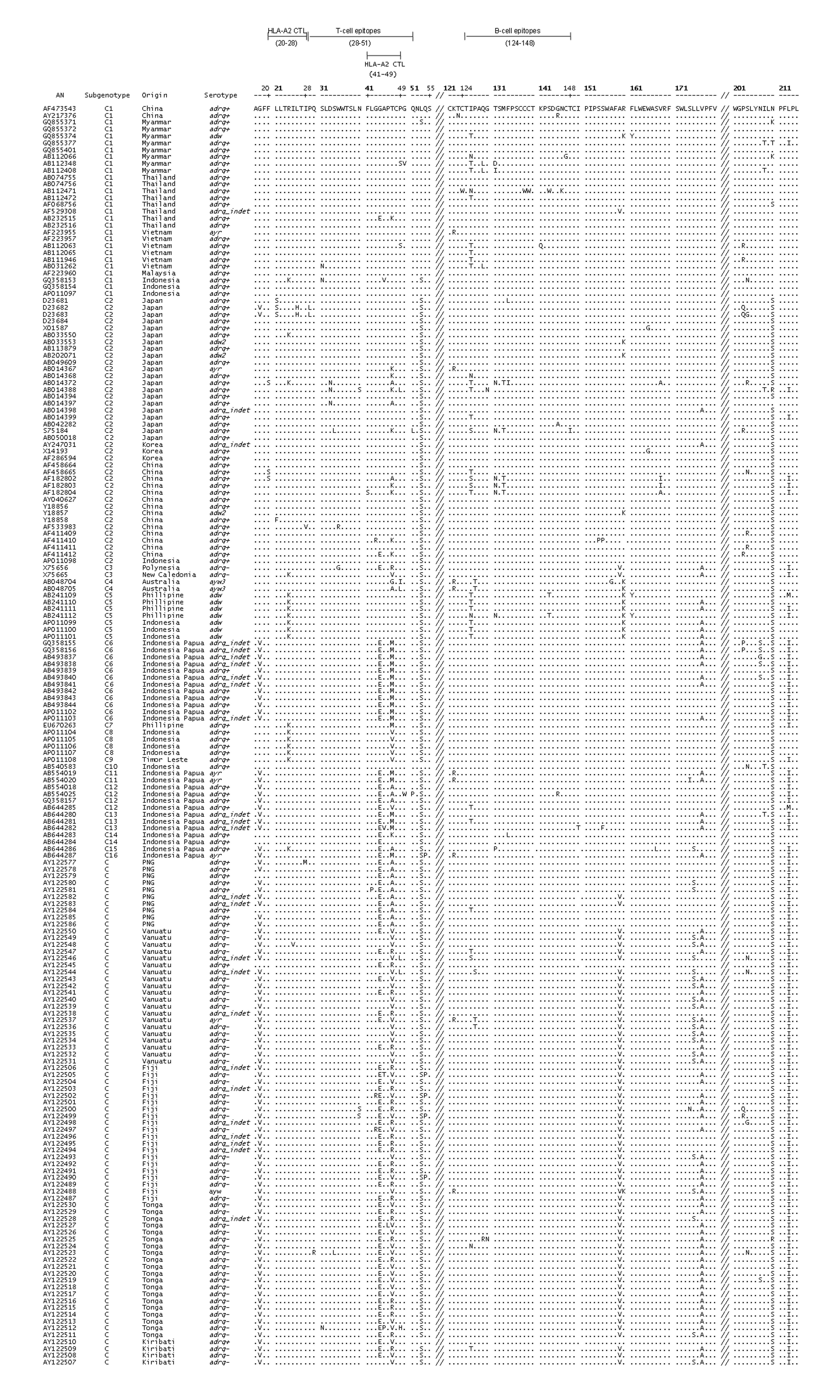

Supplement: S2 Fig — In this study, 184 isolates of various HBV/C subgenotypes were examined for immune epitopes within the surface protein (HBsAg) including the subtype-determining amino acids. The isolates were retrieved from GenBank following their origins from various geographical regions in the East and Southeast Asia and the Papua-Pacific. Variations sG18V, sG44E, and sL213I were detected in isolates from Papua-Pacific. These variations grouped all isolates into two clusters, the Asia and the Papua-Pacific (p-values <0.001; data not shown). (TIF) [file pone.0132533.s002.tif]

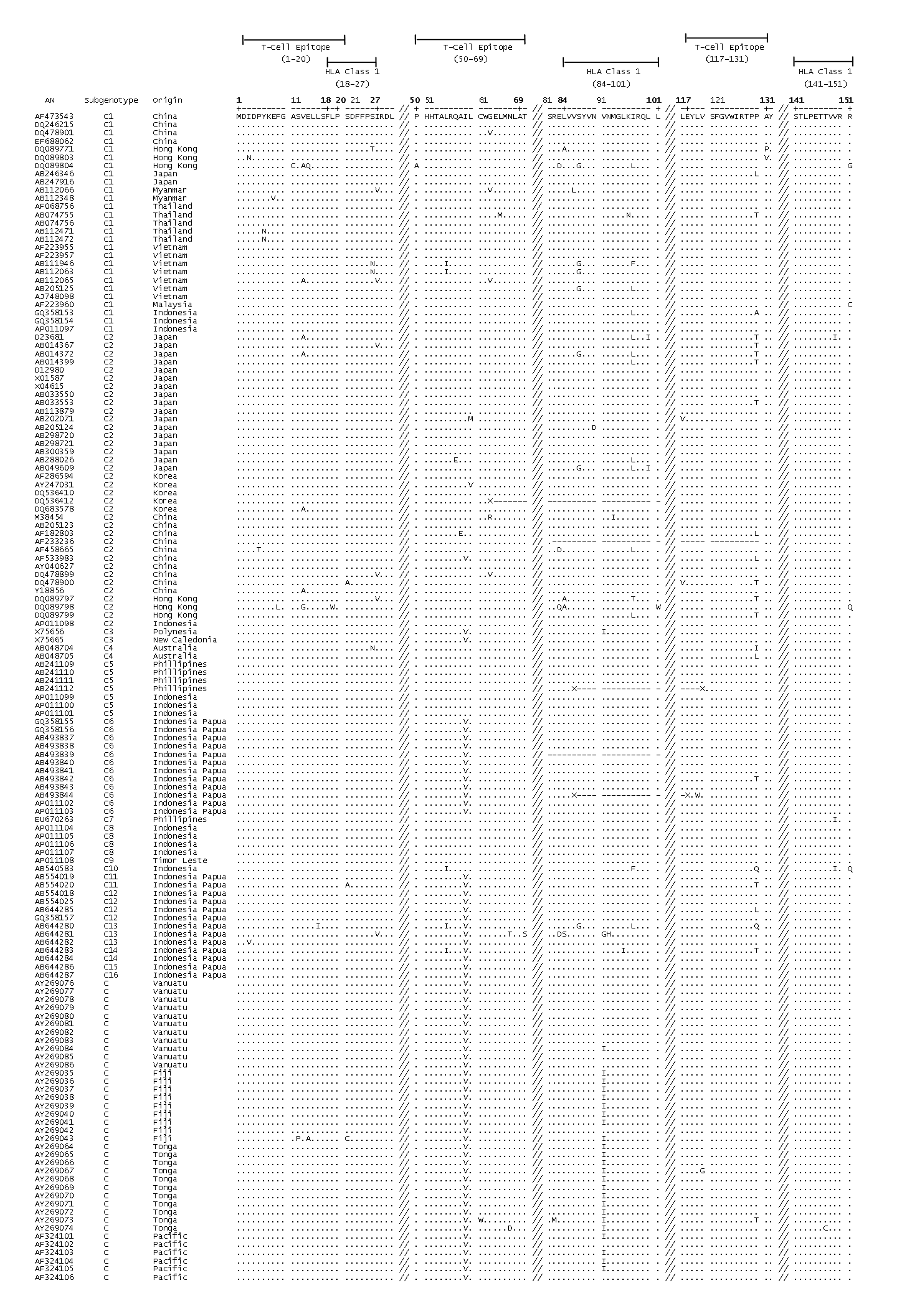

Supplement: S3 Fig — In this study, 143 isolates of various HBV/C subgenotypes were examined for immune epitopes within the core protein (HBcAg). The isolates were retrieved from GenBank following their origins from various geographical regions in the East and Southeast Asia and the Papua-Pacific. In general, compared with HBV/C isolates from Papua-Pacific, C isolates derived from Asia showed higher amino acid variation. A single amino acid variation—cI59V—markedly demonstrated the clustering of isolates from Asia and Papua-Pacific (p-value <0.001; data not shown). (TIF) [file pone.0132533.s003.tif]
