# Supplementary material for: Genogeography and Immune Epitope Characteristics of Hepatitis B Virus Genotype C Reveals Two Distinct Types: Asian and Papua-Pacific
Source: PLoS One. 2015 Jul 10;10(7):e0132533. doi: 10.1371/journal.pone.0132533 (PMC4498642; doi:10.1371/journal.pone.0132533)
Supplement: S1 Table — (DOC) [file pone.0132533.s004.doc]

**S1 Table. List of Accession Numbers of HBV sequences used in this study.**

| **No** | **Sequence group** | **Description** | | |
| --- | --- | --- | --- | --- |
|  |  | **(Sub)genotype / origin** | **Number of sequences** | **Accession Numbers** |
| 1 | 62 HBV/C complete genome | C1 | 10 | AB112066, AB112348, AB074756, AB112471, AF068756, AP011097, GQ358153, GQ358154, AF223957, AF223960 |
|  |  | C2 | 9 | AF533983, AP011098, AB033550, AB033553, AB113879, AB202071, D23681, X01587, AY247031 |
|  |  | C3 | 2 | X75656, X75665 |
|  |  | C4 | 2 | AB048704, AB048705 |
|  |  | C5 | 7 | AP011099, AP011100, AP011101, AB241109, AB241110, AB241111, AB241112 |
|  |  | C6 | 12 | AB493837, AB493838, AB493839, AB493840, AB493841, AB493842, AB493843, AB493844, AP011102, AP011103, GQ358155, GQ358156 |
|  |  | C7 | 1 | EU670263 |
|  |  | C8 | 4 | AP011104, AP011105, AP011106, AP011107 |
|  |  | C9 | 1 | AP011108 |
|  |  | C10 | 1 | AB540583 |
|  |  | C11 | 2 | AB554019, AB554020 |
|  |  | C12 | 4 | AB554018, AB554025, AB644285, GQ358157 |
|  |  | C13 | 3 | AB644280, AB644281, AB644282 |
|  |  | C14 | 2 | AB644283, AB644284 |
|  |  | C15 | 1 | AB644286 |
|  |  | C16 | 1 | AB644287 |
| 2 | 7 various HBV genotypes complete genome | A | 1 | AB126580 |
|  |  | B | 1 | AB073848 |
|  |  | D | 1 | AB090269 |
|  |  | E | 1 | AB106564 |
|  |  | F | 1 | AF223962 |
|  |  | G | 1 | AB064312 |
|  |  | H | 1 | AB266536 |
| 3 | WMHBV | WMHBV | 1 | AY226578 |
| 4 | 45 additional HBV/C complete genome | C1 | 17 | AB111946, AB112063, AB112065, AF223955, DQ246215, EF688062, AB246346, AB205125, AJ748098, DQ478901, DQ089771, AF473543, AB112472, AB074755, AB247916, DQ089803, DQ089804 |
|  |  | C2 | 25 | AB014399, DQ089799, AB288026, AF182803, AB300359, M38454, AB205123, AF233236, Y18856, DQ089797, DQ478899, DQ478900, X04615, AB049609, AB014372, AB205124, DQ683578, D12980, AF286594, DQ536410, DQ536412, AY040627, AB014367, DQ089798, AF458665 |
|  |  | C5 | 1 | AB241113 |
|  |  | C6 | 2 | GQ358153, AB493847 |
| 5 | HBV/C7, C9, C10, C15, and C16 complete genome | C7 | 1 | EU670263 |
|  |  | C9 | 1 | AP011108 |
|  |  | C10 | 1 | AB540583 |
|  |  | C15 | 1 | AB644286 |
|  |  | C16 | 1 | AB644287 |
| 6 | 48 HBV/C Asia | C1 | 18 | AF473543, AY217376, AB112408, GQ855371, GQ855372, GQ855374, GQ855377, GQ855401, AB031262, AB111946, AB112063, AB112065, AF223955, AB074755, AB112472, AB232516, AB232515, AF529308 |
|  | S gene sequence |  |  |  |
|  |  | C2 | 30 | AB014367, AB014368, AB014372, AB014388, AB014394, AB014397, AB014398, AB014399, AB042282, AB049609, AB050018, D23682, D23683, D23684, S75184, AF286594, X14153, AF182802, AF182803, AF182804, AF411409, AF411410, AF411411, AF411412, AF458664, AF458665, AY040627, Y18856, Y18857, Y18858 |
| 7 | 74 HBV/C Papua-Pacific | Papua New Guinea (PNG) | 10 | AY122577 - AY122586 |
|  |  | Vanuatu | 20 | AY122531 - AY122550 |
|  | S gene sequence | Tonga | 20 | AY122511 - AY122530 |
|  |  | Fiji | 20 | AY122487 - AY122506 |
|  |  | Kiribati | 4 | AY122507 - AY122510 |
| 8 | 87 HBV/C Indonesia |  | 87 | JQ740646 - JQ740732 |
|  | S gene sequence |  |  |  |
| 9 | 44 HBV/C Asia | C1 | 17 | AF473543, DQ246215, DQ478901, EF688062, DQ089771, DQ089803, DQ089804, AB246346, AB247916, AB074755, AB112472, AB111946, AB112063, AB112065, AB205125, AF223955, AJ748098 |
|  | C gene sequence |  |  |  |
|  |  | C2 | 27 | AB205123, AF182803, AF233236, AF458665, AY040627, DQ478899, DQ478900, M38454, Y18856, DQ089797, DQ089798, DQ089799, AB014367, AB014372, AB014399, AB049609, AB205124, AB288026, AB298720, AB298721, AB300359, D12980, X04615, AF286594, DQ536410, DQ536412, DQ683578 |
| 10 | 37 HBV/C Papua-Pacific | Fiji | 9 | AY269035 - AY269043 |
|  |  | Vanuatu | 11 | AY269076 - AY269086 |
|  | C gene sequence | Tonga | 11 | AY269064 - AY269074 |
|  |  | Pacific | 6 | AF324101 - AF324106 |
